# Medium Term Follow-Up of 337 Patients With Coronavirus Disease 2019 (COVID-19) in a Fangcang Shelter Hospital in Wuhan, China

**DOI:** 10.3389/fmed.2020.00373

**Published:** 2020-07-03

**Authors:** Nao Yan, Wei Wang, Yongzhe Gao, Junhui Zhou, Jiuhong Ye, Zhipeng Xu, Jing Cao, Junjian Zhang

**Affiliations:** ^1^Department of Neurology, Zhongnan Hospital of Wuhan University, Wuhan, China; ^2^Department of Respiratory and Critical Care Medicine, Zhongnan Hospital of Wuhan University, Wuhan, China; ^3^Wuhan East-West Lake Fangcang Shelter Hospital, Wuhan, China; ^4^Department of Nursing, Zhongnan Hospital of Wuhan University, Wuhan, China; ^5^Department of Medical Service, Zhongnan Hospital of Wuhan University, Wuhan, China

**Keywords:** COVID-19, nucleic acid, re-positive, post-discharge quarantine, discharge surveillance

## Abstract

**Background:** With the adoption of powerful preventive and therapeutic measures, a large number of patients with COVID-19 have recovered and been discharged from hospitals in Wuhan, China. Prevention of epidemic rebound is a top priority of current works. However, information regarding post-discharge quarantine and surveillance of recovered patients with COVID-19 is scarce.

**Methods:** This study followed up 337 patients with COVID-19 in a Wuhan East-West Lake Fangcang shelter hospital during the post-discharge quarantine. Demographic, clinical characteristics, comorbidities, and chest computed tomography (CT) image, mental state, medication status, and nucleic acid test data were summarized and analyzed.

**Results:** 21/337 (6.2%) patients were SARS-CoV-2 nucleic acid re-positive, and 4 /337(1.2%) patients were suspected positive. The median day interval between the discharge to nucleic acid re-positivity was 7.5 days (IQR, 6–13), ranging from 6 to 13 days. Cough/expectoration are the most common symptoms, followed by chest congestion/dyspnea during the 2 weeks post-discharge quarantine. Risk factors of nucleic acid re-positivity including the number of lobes infiltration (odds ratio[OR], 1.14; 95% CI, 1.09–1.19), distribution (OR, 0.16; 95% CI, 0.13–0.19), CT imaging feature of patchy shadowing accompanying with consolidation (OR, 9.36; 95% CI, 7.84–11.17), respiratory symptoms of cough accompanying with expectoration (OR, 1.39; 95% CI, 1.28–1.52), and chest congestion accompanying by dyspnea (OR, 1.42; 95% CI, 1.28–1.57).

**Conclusion:** The 2 weeks post-discharge quarantine may be an effective measure to prevent the outbreak from rebounding from the recovered patients. The second week is a critical period during post-discharge quarantine. Special attention should be paid to cough, expectoration, chest congestion, and dyspnea in recovered COVID-19 patients. A few recovered patients may prolong the quarantine based on clinical symptoms and signs and nucleic acid results in the 2 weeks of medical observation.

## Introduction

Since the emergence of the coronavirus disease 2019 (COVID-19), caused by severe acute respiratory syndrome coronavirus 2 (SARS-CoV-2), in Wuhan, China, in December 2019, this epidemic has caused serious harm to the health of people worldwide (1–4). On 11 March 2020, the World Health Organization (WHO) announced that COVID-19 can be characterized as a pandemic (5). As of 11 Jun 2020, 215 countries and regions have been affected by COVID-19, and there have been 7,273,958 confirmed cases of COVID-19, including 413,372 deaths, reported to the WHO.

To treat the growing numbers of COVID-19 patients in Wuhan, China, Fangcang shelter hospitals were put forward innovatively and rapidly put into practice by urgent transforming existing stadiums and exhibition centers (6). Wuhan East–West Lake Fangcang shelter hospital is the first largest Fangcang shelter hospital designated by the government, and it has provided effective treatment for a large number of patients with mild to moderate COVID-19 and has played an important role in curbing the epidemic.

With the adoption of powerful preventive and therapeutic measures, a large number of patients with COVID-19 have recovered and been discharged from hospitals in Wuhan, China. Prevention of epidemic rebound is a top priority of current works. However, information regarding clinical manifestations and discharge surveillance of recovered patients is scarce. Considering the high infectious characteristics of the SARS-CoV-2 virus, all recovered patients continue to undergo 14 days post-discharge quarantine at designated locations, which is required by the diagnosis and treatment program for novel coronavirus pneumonia (Trial Version 6).

Hence, we followed up 337 patients in Wuhan East-West Lake Fangcang shelter hospital on the 3rd, 7th, and 14th days during the post-discharge quarantine, aiming to analyze the clinical characteristics of recovered patients during the post-discharge quarantine, explore whether it is appropriate for all discharged patients to continue isolation for 14 days, and also further explore whether the 2 weeks post-discharge quarantine is an effective measure.

## Methods

### Study Design and Participants

All patients were detected to be SARS-CoV-2 nucleic acid positive by a real-time reverse transcriptase-polymerase chain reaction (RT-PCR) and classified as mild to moderate cases on admission based on the criteria issued by the National Health Commission (NHC) of the People's Republic of China. All patients were cured and discharged from Wuhan East-West Lake Fangcang shelter hospital, between 23 February 2020 and 29 February 2020, and continued isolation for 14 days at the designated location. These patients had two nucleic acid tests performed during the 2 weeks post-discharge quarantine. All nucleic acid test samples were obtained by throat swabs culture.

The discharge criteria were based on the diagnosis and treatment program for novel coronavirus pneumonia (Trial Version 6): (1) normal temperature lasting longer than 3 days, (2) resolved respiratory symptoms, (3) substantially improved acute exudative lesions on chest CT images, and (4) two consecutively negative RT-PCR test results separated by at least 1 day.

This study was approved and written informed consent was waived by the ethics committee of the Zhongnan Hospital of Wuhan University (2020075K).

### Data Collection

Demographic, clinical characteristics, chest CT Imaging, comorbidities data were extracted from patients' medical records. These patients were followed up by telephone on the 3rd, 7th, 14th days of discharge, respectively, and clinical symptoms, mental state, medication status, and the result of the nucleic acid test were detailed inquired and recorded. The final date of the follow-up was March 14, 2020.

### Statistical Analyses

Statistical analyses were done using the SPSS 22.0 software (SPSS Inc. Chicago, IL). Continuous variables were described as median (interquartile range, IQR) and compared with the Mann-Whitney U test; Categorical variables were described as a percentage and compared by χ^2^ test or Fisher's exact test. Multivariate logistic regression analysis was used to analyze the risk factors of nucleic acid positivity. A value of *P* < 0.05 was considered statistically significant.

## Results

### Demographics and Clinical Characteristics of Patients With COVID-19

Demographics and clinical characteristics are summarized in Table 1. The vast majority of patients (318/337, 94.4%) were classified as moderate patients: 19/337 patients (5.6%) were asymptomatic; 154/337 patients (45.7%) were male; and 183/337 patients (54.3%) were female. The median age for all patients was 44 years (IQR, 34–55), and 320/337 patients (94.9%) were under 65 years old. The median day of onset of symptom to hospital admission was 10 days (IQR, 7–15). A total of 221/337 patients (73.7%) were admitted within 14 days of the onset of symptoms to hospital admission. The median day of hospital stay for all patients was 17 days (IQR, 15–19), and the majority of patients have not basic diseases. A total of 43/337 patients (12.8%) have underlying comorbidity. The most common comorbidities were hypertension (14/337, 4.2%), chronic lung disease (12/337, 3.6%) including chronic bronchitis, and endocrine system disease (8/337, 2.4%) including diabetes and gout. Two patients have renal carcinoma and mammary cancer surgery history. Others including penicillin hypersensitivity, premature beat, and bilharziasis (Table 1).

**Table 1:** Demographics, clinical characteristics of patients with COVID-19.

**Demographics and clinical characteristics**	**Number (%)**
Number of patients	337
Age (years), median (IQR)	44 (34–55)
≤ 19	5 (1.5)
20–34	80 (23.7)
35–49	127 (37.7)
50–64	108 (32.0)
≥65	17 (5.1)
Gender	
Female	183 (54.3)
Male	154 (45.7)
Classification	
Mild	19 (5.6)
Moderate	318 (94.4)
The onset of symptom to hospital admission (days), median (IQR)	10 (7–15)
≤ 7	106 (31.5)
8–14	115 (34.1)
≥15	79 (23.4)
Unknown	37 (11.0)
Hospital stay (days), median (IQR)	17 (15–19)
≤ 7	9 (2.7)
8–14	73 (21.6)
15–21	241 (71.5)
≥22	14 (4.2)
Comorbidities	
Hypertension	14 (4.2)
Chronic lung disease	12 (3.6)
Endocrine system disease (including diabetes)	8 (2.4)
Cardiovascular disease	2 (0.6)
Cerebrovascular disease	2 (0.6)
Chronic kidney disease	2 (0.6)
Digestive system disease	3 (0.9)
History of tumor surgery	2 (0.6)
Others	3 (0.9)

### Chest CT Imaging Being About to Discharge

Chest CT imaging features are summarized in Table 2. Of 337 cases about to be discharged with a chest CT scan, 281/337 patients (83.4%) CT images showed that lesions were not completely absorbed after treatment; 167/337 patients (49.6%) had two lobe infiltration lesions, and this was followed by unifocal infection (23.1%), three lobes (9.5%) and four lobes (1.2%). And Additionaly, only 56/337 patients (16.6%) showed no infection lesion, and 133/337 patients (39.5%) showed multifocal infection. Ground-glass opacity (GGO) (75.1%) still was the most common CT imaging character, and this was followed by Patchy shadowing (28.2%), but the consolidation (1.5%) pattern was significantly low (Table 2).

**Table 2:** Chest CT imaging characteristics before discharge.

**Characteristics**	**Patients (*n* = 337) (%)**
**Number of lobes infiltration**	
0	56 (16.6)
1	78 (23.1)
2	167 (49.6)
3	32 (9.5)
4	4 (1.2)
5	0
**Distribution of lesions**	
Normal	56 (16.6)
Multifocal	133 (39.5)
Unifocal	148 (43.9)
**Features of lesion**	
Ground-glass opacity	253 (75.1)
Patchy shadowing	95 (28.2)
Consolidation	5 (1.5)

### Clinical Manifestations and the Nucleic Acid Test of COVID-19 Recovered Patients During Discharge Surveillance

For some reason, some patients [44/337 (13.1%), 36/337 (10.7%), and 41/337 (12.2%)] didn't complete the return visit on the 3rd, 7th, and 14th days, and 23 of these patients were lost to follow-up at all three time points. During the quarantine, 65 patients had no nucleic acid re-test results because of loss to follow-up. A total of 272 patients underwent two nucleic acid tests within 14 days. A total of 21/337 (6.2%) patients were SARS-CoV-2 nucleic acid re-positive, and 4/337 (1.2%) patients were suspected positive (Table 3). Clinical symptoms, mental state, medication status, and the result of the nucleic acid test are summarized in Table 3. Only three patients still had a fever on the 3rd day of discharge, and another patient had a fever on the 7th day of discharge, and the body temperature was only slightly elevated (37.3 or 37.4°C). Cough/expectoration (13.1%, 3rd day, 21%, 7th day, and 15.4%, 14th day) are the most common symptoms throughout the follow-up period, followed by chest congestion/dyspnea (6.6%, 3rd day, 8.6%, 7th day, and 10.4%, 14th day). Some patients also have fatigue, myalgia, sore throat, nausea, diarrhea symptoms. A few patients (2.7%, 3rd day, 3.6%, 7th day, and 4.2%, 14th day) have some other symptoms, including dizzy, wakefulness, hidrosis, headache, pectoralgia, and tinnitus. Most people have a good mental state, and very few are nervous and anxious. The majority of patients continue to be isolated and monitored in hotels or schools for 14 days, which is required by diagnosis and treatment program (Trial Version 6, 7), and a small percentage are isolated in communities and homes. During this isolation period, more than half of patients continue to be treated with Chinese traditional medicine such as novel coronavirus pneumonia No. 2 prescription, which is used to improve lung lesions. A few people also take drugs including Lianhua qingwen capsule, antiviral therapy such as oseltamivir and arbidol, and antibiotics such as quinolones and cephalosporins (Table 4).

**Table 3:** The nucleic acid test of COVID-19 recovered patients during surveillance.

**Nucleic acid re-test results**	**Patients (*n* = 337) (%)**
Re-positive	21 (6.2)
Suspected	4 (1.2)
Negative	247 (73.3)
Unknown	65 (19.3)

**Table 4:** Clinical manifestations of COVID-19 recovered patients during discharge surveillance.

**Variables**	**The 3rd day** **Number (%)**	**The 7th day** **Number (%)**	**The 14th day** **Number (%)**
Loss to follow-up	44 (13.1)	36 (10.7)	41 (12.2)
**Clinical symptoms**			
Fever	3 (0.9)	1 (0.3)	0
Cough	32 (9.5)	46 (13.6)	31 (9.2)
Expectoration	12 (3.6)	25 (7.4)	21 (6.2)
Chest congestion	15 (4.5)	19 (5.6)	28 (8.3)
Dyspnea	7 (2.1)	10 (3.0)	7 (2.1)
Fatigue	2 (0.6)	3 (0.9)	5 (1.5)
Myalgia	1 (0.3)	2 (0.6)	1 (0.3)
Sore throat	5 (1.5)	7 (2.1)	5 (1.5)
Nausea	0	1 (0.3)	1 (0.3)
Diarrhea	9 (2.7)	9 (2.7)	4 (1.2)
Others	9 (2.7)	12 (3.6)	14 (4.2)
**Mental state**			
Spirit	264 (78.3)	248 (73.6)	223 (66.2)
Full of hope	25 (7.4)	50 (14.8)	56 (16.6)
Anxiety	3 (0.3)	3 (0.9)	17 (5.0)
Depression	1 (0.3)	0	0
**Treatments in isolation areas**			
Chinese traditional medicine	105 (31.2)	205 (60.8)	196 (58.2)
Lianhua qingwen capsule	4 (1.2)	2 (0.6)	2 (0.6)
Antibiotics	1 (0.3)	5 (1.5)	1 (0.3)
Antiviral therapy	1 (0.3)	3 (0.9)	1 (0.3)
**Isolation areas**			
Hotel	177 (52.5)	176 (52.2)	136 (40.4)
School	106 (31.5)	118 (35.0)	88 (26.1)
Community	8 (2.4)	5 (1.5)	4 (1.2)
Home quarantine	2 (0.6)	2 (0.6)	52 (15.4)

### Comparisons Between Nucleic Acid Negative and Re-positive/Suspicion Patients

The median age for nucleic acid re-positive/suspicion patients was 46 years (IQR, 37–59), ranging from 20 to 65 years old, and the median day of onset of symptom to hospital admission was 11 days (IQR, 7–14.25), ranging from 7 to 14 days. The median day of hospitalization was 17 days (IQR, 16–19), ranging from 16 to 20 days. The median day interval between the discharge to nucleic acid re-positivity was 7.5 days (IQR, 6–13), ranging from 6 to 13 days. By comparing all results, we found that clinical symptoms and chest CT images were significant differences between positive/suspected positive and negative groups (*P* < 0.05) (Table 5).

**Table 5:** Comparisons between nucleic acid negative and re-positive/suspicion patients.

**Demographics and clinical characteristics**	**Negative** **Number (%) (*n* = 247)**	**Re-positive/suspicion** **Number (%) (*n* = 25)**	***P*-value**
Age (years), median (IQR)	45 (35–55)	46 (37–59)	0.553
**Gender**			
Female	136 (55.1)	15 (60.0)	0.636
Male	111 (44.9)	10 (40.0)	
**Classification**			
Mild	18 (7.3)	1 (4.0)	0.460
Moderate	229 (92.7)	24 (96.0)	
Days from illness onset (days), median (IQR)	10 (7–15)	11 (7–14.25)	0.755
Days of hospital stay (days), median (IQR)	17 (15–19)	17 (16–19)	0.418
Days of nucleic acid re-positive (days), median (IQR)		7.5 (6–13)	–
**Chest CT scan before discharge**			
Number of lobes infiltration			
0	44 (17.8)	4 (16.0)	0.017
1	54 (21.9)	4 (16.0)	
2	131 (53.0)	7 (28.0)	
3	18 (7.3)	8 (32.0)	
4	0	2 (8.0)	
5	0	0	
**Distribution**			
Normal	44 (17.8)	4 (16.0)	0.196
Unifocal	105 (42.5)	7 (28.0)	
Multifocal	98 (39.7)	14 (56.0)	
**Features of the lesion**			
Ground-glass opacity	182 (73.7)	19 (76.0)	0.802
Patchy shadowing or consolidation	73 (29.6)	14 (56.0)	0.007
**Results of follow-up**			
Clinical symptoms	80 (32.4)	13 (52.0)	0.049
Fever	4 (1.6)	1 (4.0)	0.385
Cough	57 (23.1)	8 (32.0)	0.319
Expectoration	32 (13.0)	5 (20.0)	0.501
Chest congestion	34 (13.8)	6 (24.0)	0.280
Dyspnea	18 (7.3)	2 (8.0)	0.569
**Treatments in isolation areas**			
Chinese traditional medicine	221 (89.5)	19 (76.0)	0.094
Lianhua qingwen capsule	4 (1.6)	1 (4.0)	0.385
Antibiotics	3 (1.2)	0	–
Antiviral therapy	1 (0.4)	0	–
In hospital	5 (2.0)	11 (44.0)	

Multivariate logistic regression analysis indicated the risk factors for nucleic acid positivity in recovered patients, including the number of lobe infiltrations (odds ratio[OR], 1.14; 95% CI, 1.09–1.19), distribution (OR, 0.16; 95% CI, 0.13–0.19), CT imaging features of patchy shadowing accompanying with consolidation (OR, 9.36; 95% CI, 7.84–11.17), respiratory symptoms of cough accompanying with expectoration (OR, 1.39; 95% CI, 1.28–1.52), and chest congestion accompanying with dyspnea (OR, 1.42; 95% CI, 1.28–1.57) (Table 6).

**Table 6:** Multivariable regression analysis reveal the correlations between clinical manifestations and virus nucleic acid positivity in recovered COVID-19 patients.

**Clinical characteristics**	***P*-value**	**Multivariable OR (95% CI)**
**Chest CT scan before discharge**		
Number of lobes infiltration	<0.001	2.89 (2.56–3.27)
Distribution	<0.001	0.16 (0.13–0.19)
Patchy shadowing accompanying with consolidation	<0.001	9.36 (7.84–11.17)
**Clinical symptoms**	<0.001	0.72 (0.59–0.87)
Cough accompanying with expectoration	<0.001	1.39 (1.28–1.52)
Chest congestion accompanying with dyspnea	<0.001	1.42 (1.28–1.57)

## Discussion

A recent study showed that the majority (81%) of SARS-CoV-2 nucleic acid positive patients were classified as mild cases, which displays non-pneumonia or only mild pneumonia (7). Compared with severe acute respiratory syndrome (SARS) and the Middle East respiratory syndrome (MERS), the characteristics of COVID-19 are concentrated around its significantly high infectivity (8). Thus, early isolation of the source of infection is an important measure to curb the spread of the epidemic.

With the effective prevention and control of COVID-19, a large number of patients with COVID-19 have recovered and been discharged from hospitals in Wuhan, China. However, few COVID-19 patients were nucleic acid re-positive after discharge, which caused great trouble to patients and medical staff (9, 10). This research indicates that 21/337 patients (6.2%) were SARS-CoV-2 nucleic acid re-positive, and 4/337 patients (1.2%) were suspected positive during the post-discharge quarantine. Based on actual work experience, we attribute the suspected positive patients to weak positive cases. The median day interval between the discharge to nucleic acid re-positivity was 7.5 days (IQR, 6–13), ranging from 6 to 13 days. These outcomes suggest that a proportion of recovered patients still are virus carriers, and the second week is a critical period of post-discharge quarantine. Considering that SARS-CoV-2 is a highly contagious virus, the 2 weeks of post-discharge quarantine are necessary, and this may be an effective measure to prevent the outbreak from rebounding from the recovered patients. Currently, Wuhan has just completed nucleic acid tests for nearly 9.9 million people in 16 days, and just 300 people were nucleic acid positive and classified as asymptomatic, the detection rate was about 30.3/million. The result to some extent certified the reliability of post-discharge quarantine.

During the post-discharge quarantine, a small number of recovered patients experienced a recurrence of these clinical symptoms. Cough or expectoration is the most common symptom in patients with SARS-CoV-2 nucleic acid re-positive. What's more, logistic regression analysis found that cough accompanying with expectoration and chest congestion accompanying with dyspnea are the risk factors for nucleic acid re-positivity in recovered patients with COVID-19. The result indicates that the risk of nucleic acid re-positivity increased by 1.39 and 1.42 times, respectively. Thus, these patients with respiratory symptoms should be paid more attention to, and nucleic acid testing and chest CT should be conducted during the 2 weeks of discharge surveillance. These patients may prolong the discharge quarantine based on clinical symptoms and signs and nucleic acid results.

In these discharged patients, we found that Ground-glass opacity (GGO) (75.1%) was still the most common CT imaging, but the imaging feature of consolidation (1.5%) was significantly low. Considering that the lung lesions are not fully absorbed, most discharged patients continue to be treated with medication such as Chinese traditional medicine, Lianhua Qingwen, antivirals, etc. Although there are no specific therapeutic drugs and vaccines, basic therapy, such as antiviral therapy and oxygen inhalation, still had some effect, which prevents the consolidation of lung lesions. Indeed, consolidation pattern is considered as an indication of disease progression and more occurs in severe and critical COVID-19 cases (11, 12). Even so, by analyzing re-positivity or suspicion patient's CT image characteristics, we still found that the number of lobe infiltrations, distribution, and patchy shadowing accompanying with consolidation are the risk factors for nucleic acid re-positivity in recovered patients. The result indicates that the risk of nucleic acid re-positivity increased by 2.89, 0.16, and 9.36 times, respectively. Given the situation of recovered patients' nucleic acid re-positive, individualized discharge criteria should be formulated according to the factors of age, comorbidities, clinical symptoms, chest CT image, and degree of illness.

COVID-19 is a new infectious disease. Genomic analysis has revealed that SARS-CoV-2 is a member of the *Betacoronavirus* genus that includes SARS-CoV, MERS-CoV, and SARS-CoV-2 shares a highly homological sequence with Bat coronavirus RaTG13 (with 93.1% in the spike gene). Recent studies confirm that the angiotensin-converting enzyme 2 (ACE2) receptor is a critical site for SARS-CoV to infects host cells (13, 14). The ACE2 protein expresses in multiple human organs including lung, small intestine, colon, liver, kidney, and brain, and SARS-CoV-2 can also invade multiple human systems. Thus, the virus may harbor in other organs such as the intestine when the respiratory tract virus was cleared, and throat swab nucleic acid testing was negative at the late stage of treatment (15, 16). There are several other possible reasons for SARS-CoV-2 nucleic acid re-positivity. First, a false negative is possible due to RT-PCR detection sensitivity and limitations of throat swab sample collection (17, 18). Second, the low viral load wasn't enough to be detected since the virus be suppressed after receiving treatment during hospitalization; the residual viral genome could thus continue to proliferate and be restored to a detectable level during the quarantine. A recent study shows that viral loads are higher at the initial stage and reach the highest level in the second week from the onset of symptoms in mild patients (19). Third, the underlying comorbidities, clinical status, glucocorticoid use, and the biological characteristics of SARS-CoV-2 might be related to the process of nucleic acid re-positivity (20). Nevertheless, the exact molecular mechanism of nucleic acid re-positivity needs further study.

The study also has some limitations, this is a single-center study and the sample size is relatively small; nevertheless, all fangcang hospitals followed uniform admission and discharge criteria issued by the National Health Commission (NHC) of the People's Republic of China. The clinical characteristics of 337 patients were similar to hospitalized patients in some published studies (2, 3). The study findings should supply important information regarding recovered patients post-discharge quarantine and surveillance. Additionally, the 2 weeks post-discharge quarantine and medical observation are an essential measure to prevent the outbreak from rebounding.

In conclusion, the 2 weeks post-discharge quarantine may be an effective measure to prevent the outbreak from rebounding from the recovered patients. The second week is a critical period of post-discharge quarantine. Cough, expectoration, chest congestion, and dyspnea should be paid special attention for recovered patients, and nucleic acid testing and chest CT should be conducted in time during discharge surveillance. A few recovered patients may prolong the quarantine based on clinical symptoms and signs and nucleic acid results in the 2 weeks of medical observation.

## Data Availability Statement

All datasets presented in this study are included in the article/supplementary material.

## Ethics Statement

This study was approved and written informed consent was waived by the Ethics Committee of the Zhongnan Hospital of Wuhan University (2020090K).

## Author Contributions

NY and WW performed all the data analysis and wrote the first draft of the manuscript. JZha provided important clinical content to the manuscript and supervised the study. YG and JZho contributed to the critical revision of the manuscript for important intellectual content. JY, ZX, and JC provided administrative, technical, and material support. All authors contributed to the article and approved the submitted version.

## Conflict of Interest

The authors declare that the research was conducted in the absence of any commercial or financial relationships that could be construed as a potential conflict of interest.
